# Taking stock of the social determinants of health: A scoping review

**DOI:** 10.1371/journal.pone.0177306

**Published:** 2017-05-11

**Authors:** Kelsey Lucyk, Lindsay McLaren

**Affiliations:** Department of Community Health Sciences, Cumming School of Medicine, University of Calgary, Calgary, Alberta, Canada; University of South Carolina, UNITED STATES

## Abstract

**Background:**

In recent decades, the social determinants of health (SDOH) has gained increasing prominence as a foundational concept for population and public health in academic literature and policy documents, internationally. However, alongside its widespread dissemination, and in light of multiple conceptual models, lists, and frameworks, some dilution and confusion is apparent. This scoping review represents an attempt to take stock of SDOH literature in the context of contemporary population and public health.

**Methods:**

We conducted a scoping review to synthesize and map SDOH literature, informed by the methods of Arksey and O’Malley (2005). We searched 5 academic and 3 grey literature databases for “social determinants of health” and “population health” or “public health” or “health promotion,” published 2004–2014. We also conducted a search on “inequity” or “inequality” or “disparity” or “social gradient” and “Canad*” to ensure that we captured articles where this language was used to discuss the SDOH. We included articles that discussed SDOH in depth, either explicitly or in implicit but nuanced ways. We hand-searched reference lists to further identify relevant articles.

**Findings:**

Our synthesis of 108 articles showed wide variation by study setting, target audience, and geographic scope, with most articles published in an academic setting, by Canadian authors, for policy-maker audiences. SDOH were communicated by authors as a list, model, or story; each with strengths and weaknesses. Thematic analysis identified one theme: health equity as an overarching and binding concept to the SDOH. Health equity was understood in different ways with implications for action on the SDOH.

**Conclusions:**

Among the vast SDOH literature, there is a need to identify and clearly articulate the essence and implications of the SDOH concept. We recommend that authors be intentional in their efforts to present and discuss SDOH to ensure that they speak to its foundational concept of health equity.

## Introduction

### Overview

In recent decades, the social determinants of health (SDOH), that is the social, economic, and political conditions that influence the health of individuals and populations, has gained increasing prominence as a foundational concept to the field of population and public health (PPH). During the past 15 years, the SDOH concept has evolved to the point of being a formal component of many undergraduate and graduate training programs in PPH and related fields, and thus it is timely to take stock of the SDOH literature and identify its major themes in this context.

### Background

In this paper, we use the term “population and public health” to refer to the shared goals, combined efforts, and overlapping histories of population health and public health. Public health refers to the organized and collective efforts of society (e.g., health promotion, disease prevention, emergency preparedness, health protection)[1] to assure conditions for people to be healthy. Population health is an approach that studies disease burdens, risks, determinants, vulnerabilities, and conditions (e.g., of living and working) among population groups with the aim of reducing health inequities through action on the structural influences (e.g., SDOH).[2–4] The combined field of PPH research and practice therefore includes multiple actors and agencies in governmental and academic spheres of influence, as well as the voluntary and private sectors.

In Canadian and United Kingdom (UK) policy circles, the SDOH concept has been increasingly incorporated into PPH literature and policies since it first gained recognition in the 1970s and 1980s alongside health promotion (i.e., the process of enabling people to increase control over and improve their health.[5]) When considered together, the uptake of SDOH and health promotion by the PPH community represents a shift away from a focus on the individual-level factors that influence health, towards factors at the community and societal levels. Some prominent early examples of the SDOH concept, before it was named as such, appear in health policy documents such as the Canadian *Lalonde Report*[6] in 1974 and the UK *Black Report*[7] in 1980. The 1974 *Lalonde Report*, known formally as *A New Perspective on the Health of Canadians*,[6] represents the first government document in the Western world to acknowledge factors external to the health care system in achieving health (e.g., environment, lifestyle).[8] In the UK, the 1980 Black Report–*Report of the Working Group on Inequalities in Health*–found that inequities in health between upper and lower classes persisted despite universal access to health care.[9] Aside from policy documents, the SDOH has also gained prominence in the academic literature through studies that elucidated findings on concepts such as the social gradient in health. The Whitehall Studies conducted by Marmot and colleagues throughout the late 1970s and 1980s illustrated this stepwise relationship regarding mortality rates among different employment grades of British civil servants, which were used as a measure of social class.[10–11]

Because the SDOH concept is multifaceted, different models and theories have emerged in the literature to try and explain what the SDOH are, how they operate, and how they can be addressed via policy. Examples of these models include: the life course model, the allostatic load model, theories of materialism and neomaterialism, and population health promotion theory.[9,12–22] As described in detail later in this paper, some of these models privilege more ‘downstream’ efforts to increase access to health and social services or resources at the individual or family level, while others represent more ‘upstream’ efforts to reform the distribution of power, wealth, opportunities, and decision-making at the societal level.[23] The many theoretical models and ways the SDOH are operationalized have created “conceptual ambiguity.”[24] When faced with this ambiguity, students, researchers, policy-makers, or members of the general public who are new to the SDOH concept may find it difficult to extract the key messages.[25] Considering the ongoing efforts to approach SDOH from an intersectoral and multidisciplinary perspective,[26] a clear understanding of the SDOH concept is especially important. Thus, there remains the need to discern key components from the SDOH concept, which is the purpose of this paper.

Recent attempts have been made to synthesize literature on the SDOH. Some examples include the body of work by Raphael and colleagues,[27–35] and contributions from the World Health Organization’s (WHO) Commission on the Social Determinants of Health (CSDH).[16–17] These documents are important to the SDOH literature, as they have helped strengthen the theoretical basis of the field, yet they do have some limitations. First, previous syntheses have not been explicitly systematic. Second, the time period for many of these contributions predate the 2008 WHO CSDH, which brought significant public attention to the SDOH, as reported by a recent media analysis on the coverage of SDOH in print news media.[36] The significance of the WHO CSDH to the SDOH field warrants revisiting the literature contemporarily. Finally, prior literature reviews of the SDOH have focused on specific health conditions,[37–40] health services,[41–43] populations,[43–47] or theories (e.g., policy analysis theory, systems change),[48–49] and not the concept as a whole.

This paper reports on findings from a scoping review of SDOH-related academic and grey literature from the fields of population health, public health, and health promotion. Our purpose is to discern key concepts and themes about the SDOH as evidenced in the PPH literature. The novelty of this review lies in our comprehensive and multidisciplinary perspective and inclusion of grey literature. We explicitly focus on the concept of SDOH as a whole, rather than its contributive role to narrower topics (i.e., specific health conditions). Additionally, by situating our work within the broad, overlapping scholarly and applied fields of population health, public health, and health promotion, we cast a wide net in our search strategy which (to the best of our knowledge) has not been done. Our approach allows for reflection on the current state of the SDOH with recognition of health promotion’s historic influences on this concept’s development. Finally, the time frame of our review allows for the consideration of articles that represent more recent contributions to the SDOH literature (e.g., since the WHO CSDH 2008 report).

This review will be of interest to those working and studying in population health, public health, and health promotion. Specifically, it may serve as a resource for students looking to navigate this vast and complex field, as well as scholars from various disciplines who wish to situate themselves within the foundations of PPH.

## Methods

We conducted a scoping review to synthesize and map literature about the SDOH within the scope of PPH. We followed the framework outlined by Arksey and O’Malley (2005)[50] in their methodological paper on scoping studies and by drawing on the methods of two recent publications.[51–52] Scoping review studies differ from systematic reviews in their breadth and aims.[50] Systematic reviews tend to ask more narrowly-defined questions and answer these questions from a narrower range of studies that have been formally appraised for quality.[50] Scoping reviews ask broader questions and do not assess the quality of studies reviewed.[50] Scoping reviews may be undertaken to examine the range and extent of research on a topic, summarize and disseminate findings, identify gaps in the literature, or to determine the value of a conducting a systematic review.[50] Our aim was to summarize and disseminate findings. Specifically, we sought to answer the research question: what are the key terms, concepts, and ideas associated with the social determinants of health within PPH? We adopted a comprehensive approach because our findings are intended to inform another study in progress, which aims to trace the evolution of the SDOH concept (as identified through this review) in contemporary Canadian history.

### Analysis

We extracted information related to each study’s location (i.e., based on the first author’s institutional affiliation), audience (i.e., implied based on the paper’s purpose and recommendations), date of publication, and setting (i.e., based on the geographic location of the first author’s affiliation) to understand the landscape of the literature. Next, using NVivo QSR software,[53] we coded and organized the documents and generated themes. We first coded all studies for ideas, terms, and concepts that emerged repeatedly in the literature.[54] Then, we developed themes iteratively by rereading our sources, reviewing our codes, and identifying patterns in the data.[55]

We also conducted a quantitative content analysis of the key concepts identified from thematic analysis by calculating the proportion of articles that included key terms. Both our qualitative and quantitative analyses were informed by the methodology for content analysis described by Krippendorff (2004), which regards texts as meaningful representations of human phenomena.[54] Content analysts, through asking questions, interpreting, and closely reading texts, infer meaning from the common components, patterns, or trends they observe.[54] Following Krippendorff’s (2004) steps we recorded information from and coded information about our texts, tabulated our findings to determine how frequently these words appeared in the literature, and also interpreted them narratively.[54] Finally, we grouped our codes and key concepts into wider themes that synthesized the literature as a whole, which we explore in-depth below.

### Search and inclusion/exclusion strategy

We searched 5 academic (CINAHL, Embase, Medline, PsycInfo, PubMed, SocIndex) and 3 grey literature databases (Google [general], Canadian Health Research Collection, Canadian Research Index) for the terms “social determinants of health” and (“public health” or “population health” or “health promotion”) in the article’s subject heading, title, abstract, or keyword section. We limited our search to those with English language abstracts published between 2004 and 2014.

**Fig 1:**
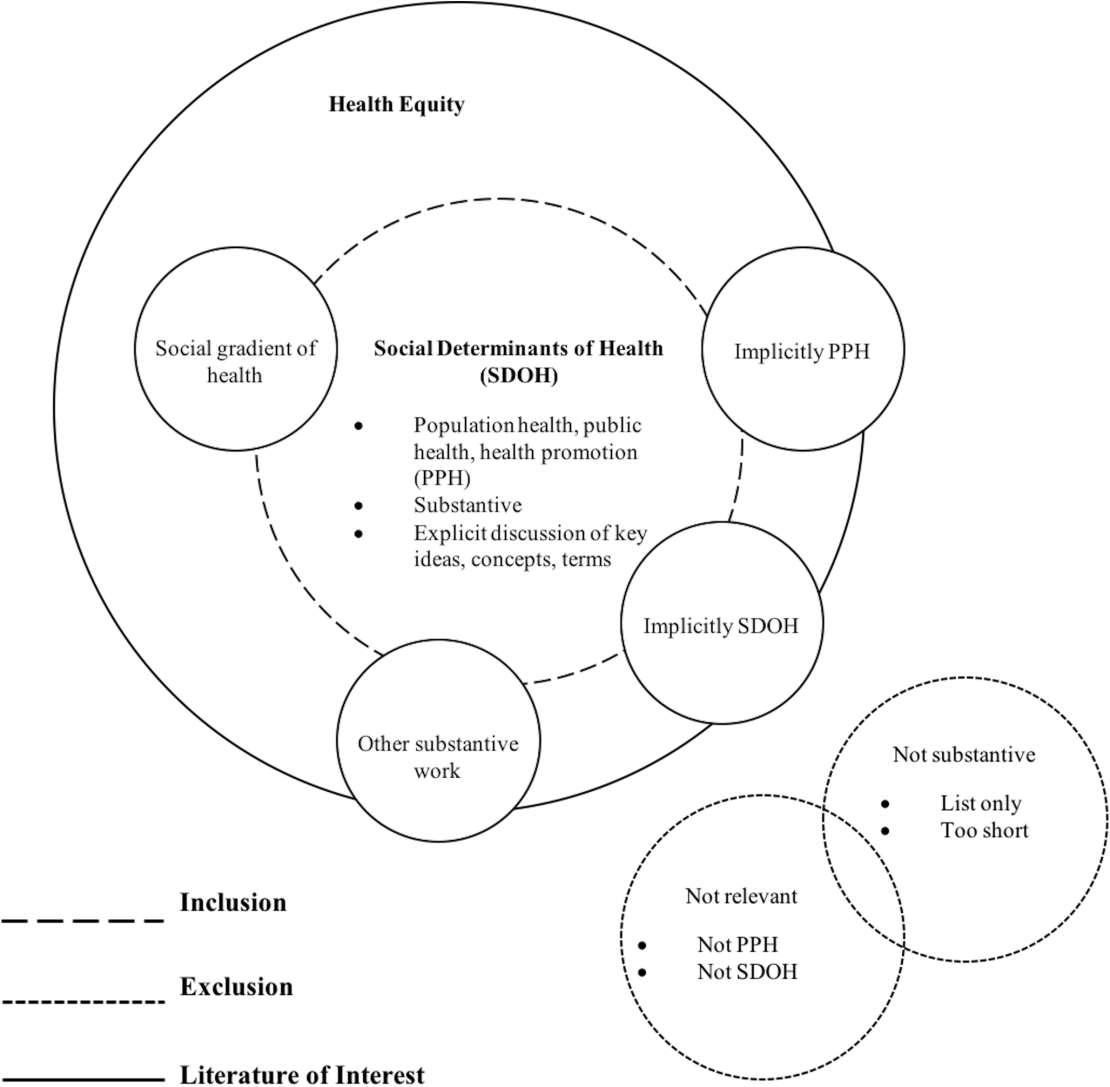
Visual representation of approach to inclusion criteria. The conceptual search strategy used to capture various bodies of literature.

Before commencing this review, we understood that the SDOH literature contained a wide variety of document styles, including papers that list or mention the SDOH without any elaboration, as well as papers that provide substantive discussion. Because we were interested in the latter, our approach to identifying those materials was by necessity iterative and flexible. We privileged articles that contained explicit discussion of the SDOH and its related ideas, concepts, or key terms, and excluded articles that did not. To be included, articles had to go beyond description to consider the ‘why’ and ‘how’ elements of the SDOH. As we gained familiarity with the literature, we purposively began to include articles that discussed the SDOH in more nuanced ways (e.g., social gradient, inequities, social factors), regardless of whether they explicitly mentioned ‘SDOH.’ We privileged papers that were, in our view, clearly about PPH regardless of whether that was explicitly mentioned. We also searched reference lists of articles to further identify articles that were pertinent but not captured by the parameters of our search (i.e., papers that were well-known and widely consulted in the PPH community were always considered for possible inclusion). Overall, as befits the nature of the field, we used a more flexible approach to inclusion/exclusion (see Fig 1. Visual representation of approach to inclusion criteria) than one might find in reviews of other subject areas.

Authors developed the inclusion and exclusion criteria collaboratively, and revised them as we gained familiarity with the literature. KL applied the inclusion/exclusion criteria to all titles and abstracts, after which LM reviewed the selections. Both authors agreed that the sample of articles selected for full-text review, described below, were relevant to our research question. KL extracted data from relevant articles and met regularly with LM to discuss findings. It was during these meetings that key concepts were discerned and themes were generated.

Early in our title and abstract review, we recognized that the concept of health inequity was used repeatedly in the literature in ways akin to our understanding of the SDOH, even in articles where the SDOH were not explicitly mentioned. Braveman et al. (2011), for example, discussed health disparities and health equity in their abstract, but went on to discuss the SDOH in detail in the full-text of their article.[56] Therefore we felt it necessary to revisit our search strategy to ensure that we were capturing such articles. We first tested the feasibility of a revised search in PubMed, by adding the terms “inequity” or “inequality” or “disparity” or “social gradient” to our search. With no geographic parameters, this returned 28,472 abstracts, which we deemed insufficiently sensitive and not feasible for this review. We then tried restricting the geographic scope by adding the term “Canad*”. As described below in detail, this resulted in the identification of 619 abstracts, which were incorporated into our review. The implications of this Canadian-specific inequity search are discussed in the limitations section of our paper.

## Results

We present our results as follows. First, we introduce our descriptive findings of the literature regarding number of articles published by date, institutional affiliation of the first author, target audience, and geographic setting. Second, we explore the different ways that the literature presented the SDOH as a list, model, or story. We consider the implications of these different presentations and show they may align with different epistemologies. Third, we discuss health equity as a key theme and binding concept of the SDOH and explore how it, and the related concept of the social gradient in health, have been used in the literature. Finally, we demonstrate how action on the SDOH has been conceptualized in the literature, through more ‘upstream’ or ‘downstream’ approaches.

### Descriptive findings

**Fig 2:**
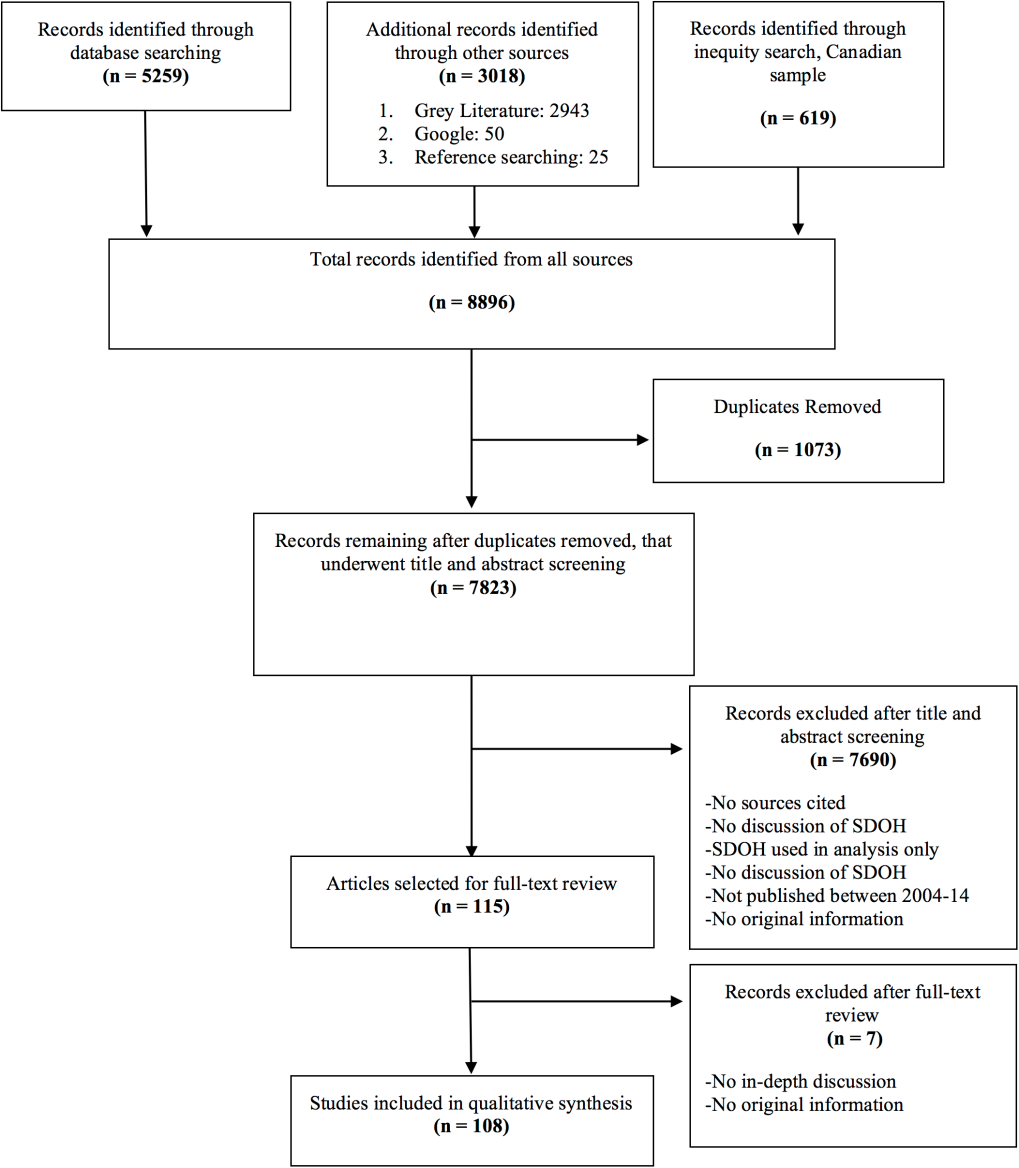
Flow diagram for search of SDOH literature for exclusion reasons. PRISMA diagram showing search and selection process of literature review.

Our initial search returned 5259 articles from our database search, 3018 articles from grey literature, Google, and reference lists, and 619 articles from our Canadian inequity search, for a total sample of 8896 articles. After duplicates were removed, 7823 articles remained and underwent title and abstract review for relevance. During this stage, we excluded 7708 articles (see Fig 2. Flow diagram for search of SDOH literature for exclusion reasons) and retained 115 articles for full-text review. We excluded 7 articles during our full-text review (see Fig 2. Flow diagram for search of SDOH literature for exclusion reasons) and retained 108 articles for qualitative synthesis. A summary of our review process is shown in Fig 2. Flow diagram for search of SDOH literature (PRISMA).

### Time trends and the impact of the WHO CSDH

Of the time period considered (1986 to 2014), the most active period in terms of numbers of publications was 2005 to 2009 for grey literature (with 46.9% of included grey literature documents being published during that period) and 2010 to 2014 for academic publications (with 43.4% of included academic publications being published during that period). Time trends are summarized in Table 1.

**Table 1:** Descriptive characteristics of SDOH literature.

	All Articles (N = 108)		Grey literature* (n = 32)		Academic literature * (n = 76)		Canadian Sample Only (n = 5)	
Characteristic	No.	%	No	%	No.	%	No.	%
*Publication date*								
Before 2000	6	5.6	2	6.3	4	5.3	0	0
2000–2004	9	8.3	2	6.3	7	9.2	0	0
2005–2009	45	41.7	15	46.9	30	39.5	2	40.0
2010–2014	43	39.8	10	31.3	33	43.4	3	60.0
No date (webpages)	5	4.6	3	9.4	2	2.6	0	0
*First Author Institutional Affiliation*								
Academic	76	70.4	0	0	76	100.0	5	100.0
Non-academic	32	29.6	32	100	0	0	0	0
*Study Location (derived from first author’s location if none specified)*								
Canada	57	52.8	23	71.9	33	43.4	5	100.0
United States	20	18.5	2	6.3	18	23.7	0	0
Australia	5	4.6	0	0	5	6.7	0	0
UK	15	13.9	1	3.1	14	18.4	0	0
Germany	2	1.9	0	0	2	2.6	0	0
Spain	2	1.9	0	0	2	2.6	0	0
Sweden	1	0.9	0	0	1	1.3	0	0
Switzerland (WHO)	6	5.6	6	18.8	1	1.3	0	0
*Target Audience/End Users*								
Academia	14	13.0	0	0	14	18.4	0	0
Public Health Workforce	37	34.3	12	37.5	25	32.9	2	40.0
Policy	45	41.7	18	56.3	27	35.5	1	20.0
Academia and Policy	3	2.8	0	0	3	3.9	1	20.0
Academia and Public Health Workforce	2	1.9	0	0	2	2.6	0	0
Academic, Public Health Workforce, and Policy	1	0.9	0	0	1	1.3	0	0
Public Health Workforce and Policy	6	5.6	2	6.3	4	5.3	1	20.0

*The categories “academic” and “grey” literature were applied to articles during the review phase and do not necessarily reflect the database that returned them. This was done to overcome instances where academic articles appeared in the grey literature.

The large numbers of documents appearing in the 2005 to 2009 and 2010 to 2014 periods (relative to the prior periods) likely reflects the momentum of the WHO CSDH, which commenced in 2005 and culminated with its final report in 2008.[17] Many of our included articles used the WHO CSDH to frame their work and/or support its timeliness and relevance.[57–64] Other articles built on the work of the WHO CSDH to contribute to the literature for certain populations, such as articles that discussed SDOH specific to the Métis population, British Columbians, or populations in conflict settings.[65–68] Some articles sought to critique the work or scope of the WHO CSDH,[69–70] reflect on its process,[71] or respond to its findings.[72] Others simply included the WHO CSDH in their work by adopting its framework and reiterating its goals.[73–74] Some articles that were published prior to the WHO CSDH report (e.g., in the mid-2000s) highlighted the anticipated contributions of that initiative.[16,75–77]

### First author institutional affiliation of SDOH literature

We assigned each article a study setting, based on the first author’s institutional affiliation. We identified two types of settings: academic (e.g., university professor, fellow, student), and non-academic (e.g., government ministry or department, non-profit organization, or regional health authorities). We recognize that these categories may at times overlap, especially where authors collaborate with co-authors from other institutional settings or where authors have multiple affiliations but publish only under a certain one (e.g., a government employee publishing under their academic affiliation). However, these categories do provide insight into the different sectors involved in producing and contributing to SDOH literature.

The majority of articles in this review (70.4%) were published in an academic setting by authors who were affiliated with health research (Table 1). Authors came from a variety of academic disciplines that included health-related disciplines such as public health,[64,78] population health,[26] nursing,[71] medicine,[79] social work,[80] epidemiology,[81] and social science disciplines, such as sociology,[9,69] sociomedical or social sciences,[21,33,82] geography,[18] governance,[83] social policy,[84] communication,[85] and economics.[22]

The rest of the articles (29.6%) were published by individuals or groups working in or affiliated with government departments and ministries, non-profit or professional organizations, or health authorities (Table 1). Some publications were authored by representatives of government organizations, such as the UK National Health Service,[86] Chief Medical Officer of Health,[87] Public Health Agency of Canada,[15] Health Canada,[88] or the United States (US) Department of Health and Human Services.[89] Other articles were authored by professional associations, such as the Canadian Public Health Association,[90] Canadian Nurses Association,[91] Canadian Medical Association,[92] or the Health Officers Council of British Columbia.[65] Others still were authored by practitioners from health authorities, such as Alberta Health Services[93]), or by members of knowledge translation groups, such as the National Collaborating Centre for Aboriginal Health.[84]

Overall, the SDOH literature in the context of contemporary PPH is shown to be widely interdisciplinary and produced by those in both academic and professional/applied settings.

### Implied target audience of SDOH literature

In most cases, authors were not explicit about their audience of interest. Therefore, for each article, we identified what seemed to be the implicit target audience, based on the paper’s purpose and recommendations (e.g., to increase understanding of something versus to make recommendations for government action), and the level of discussion (e.g., plain language versus complex theoretical concepts). We grouped implicit target audiences into three categories: academics, policy and decision-makers, and the public health workforce (Table 1).

Many of the articles (41.7%) seem to have been written with the intent of reaching policy and decision-makers (Table 1). Most of these came from the academic literature (n = 27), though some came from the grey literature (n = 18). An article by O’Campo (2012), for example, concluded that authors should focus on synthesizing evidence on multilevel determinants of health and evaluating macro-social policies and programs so the evidence may be of use to policy makers.[94] Other articles (34.3%) were written for the public health workforce (e.g., practitioners, public health physicians, nurses, etc.). One example is the Canadian Nurses Association backgrounder considering the role for nurses in acting on the SDOH.[91] Articles were mostly academic (n = 25), though some were also from grey literature (n = 12).

Finally, some articles (13.0%) appeared to be directed at an academic audience. These articles all came from the academic literature (n = 14). Examples include those written for the purpose of research methodological clarification (e.g., Regidor’s review of measurements of socioeconomic position),[24] or articles that presented theoretical positions (e.g., Link and Phelan’s theory of fundamental causes[95] or Tarlov’s theoretical work on the sociobiological translation[96]). Many articles were targeted to more than one audience.

### Geographic setting of SDOH literature

We assigned articles a geographic setting based on the location of the first author’s institutional affiliation. Just over half (52.8%) of our included publications were from Canada, followed by the US (18.5%) and the UK (13.9%) (Table 1). For the grey literature, an even higher proportion (71.9%) was from Canada. This was true for academic and grey literature, as well as for the studies that came from our search targeting Canadian literature on inequity (i.e., the “Canadian Sample Only” column in Table 1). This finding may speak to professor Dennis Raphael’s observation that Canada has an international reputation as a ‘powerhouse’ based its written contributions to health promotion, population health, and the SDOH.[35,62,97–98] However, as Manzano and Raphael (2010) have pointed out, these written contributions have not been accompanied by substantive action, and in fact Canada has increasingly weakened its commitments to improving the SDOH and health equity.[62] The many documents published by Canadian government organizations and scholars perhaps supports the claim that Canada is a document powerhouse that falls short in acting on the SDOH (e.g.,[15, 20, 22, 27–30, 33, 57, 62–63, 97–102]).

### Different ways of presenting and communicating the SDOH

We observed differences in the ways that SDOH were presented and communicated as a list, model, or story. Our purpose is not to evaluate these various approaches but to document their existence, features, and purposes within the literature. While we reflect on the various approaches, we intentionally do not evaluate them in part due to our inclusion criteria, which prioritized sources that took a more narrative approach to the SDOH.

#### Communicating the SDOH as a list of influential factors

The list approach to organizing and communicating the SDOH has benefits and challenges. One benefit is that lists present information about the SDOH in organized ways that highlight important features to readers. This may facilitate dissemination and widespread understanding, where listed points are clear, concise, and easily reproducible (e.g., make a photocopy to share with colleagues). A challenge of communicating the SDOH in lists, however, is that lists are not exhaustive, as authors must choose what information is included. Additionally, lists may lead to confusion where many versions exist on the same topic (see, for example, Raphael’s [2006] article which compares Canadian SDOH lists).[99] Some lists attempt to be as comprehensive as possible within the scope of their work, for example by compiling a glossary to accompany listed terms,[74] while others prioritize certain SDOH over others for different audiences or topics of interest.[34, 75, 81] Additionally, authors do not always state their intentions in compiling SDOH lists, which may have implications for how their lists are interpreted. Some lists, for example, may be purposefully organized to list the highest priority SDOH first, while others may be less intentionally compiled (e.g., alphabetical order). Important information may be buried in this case, should readers uncritically skim lists from top to bottom. Alternatively, readers may assign greater importance to the listed elements they read first. To some extent, the above challenges could be addressed if a single list was adopted by authors. Raphael’s list SDOH represents one example that has been widely adopted by Canadian authors.[34]

The list approach also presents a potential challenge for communicating the complexity of the SDOH. The SDOH represent much more than a list can convey, such as issues related to how listed SDOH intersect with one another, the social and historical nature of SDOH, or the foundational role of equity. With lists, there is also the drawback of being too inclusive or providing too much breadth to be of practical use. An overly inclusive list does not provide direction, and may direct focus to issues that are at the periphery of the SDOH, perhaps because they are or seem to be the easiest to address. However, lists do serve the needs of many authors, especially those who wish to briefly communicate pertinent elements of the SDOH to their audience. This may be especially true among grey literature publications, for example where SDOH resources are produced to inform practitioners. Academics, on the other hand, may publish as an opportunity to theoretically interrogate or expand upon the SDOH, taking a more narrative approach.

As indicated by its name, the list approach to the SDOH provides a list of factors that influence health. The British Columbia Government, for example, in their 2008 discussion paper on health inequities in the province provides readers with two lists of the SDOH–one noting upstream influences (i.e., macroeconomic policies; culture, ethnicity and values; governance; income and social status; employment and working conditions; education and literacy; and, early childhood development) and the other downstream determinants (i.e., physical built environments; social support networks; social environments; access to effective health care services; risk behaviours; personal health practices and coping skills; gender; and, biological and genetic endowment).[65] The elements of these lists appear to have been purposively chosen to expand to the discussion of policy options for action on the SDOH among a wide range of audiences (e.g., health professionals, decision makers). We discuss the notions of upstream and downstream interventions in detail later.

#### Communicating the SDOH through conceptual models

The model, or conceptual framework, approach moves beyond a list of SDOH to show (often visually) how various elements interconnect and are experienced at different levels (e.g., societal, community, family, individual) to produce different outcomes (e.g., opportunities, health outcomes, distribution of opportunities). Most models share the idea that health represents a web of social influences.[103] Well-known examples of SDOH models, presented in chronological order, include Evans and Stoddart’s (1990) framework, which shows how individual and social factors interact outside of the health care system,[22] Whitehead and Dahlgren’s (1991) ‘rainbow model’ which shows concentric half-circles of influential social factors,[104] and more recently, Solar and Irwin’s (2007) conceptual model produced for the WHO that shows the multiple directions through which structural and intermediary determinants impact health and health equity.[16] Lesser known examples include Fox and Meier’s (2009) right to development SDOH model [21] and the model for Métis SDOH that shows interrelationships specific to this population (e.g., self-determination, land, colonization).[66] While numerous other models exist, they have been documented elsewhere [e.g., [20, 89, 93, 103, 105–106]] and will not be reviewed here. A comprehensive and illustrative guide to various models of the SDOH, including those outside the scope of this review, is provided in MacDowell’s webpage created for medical students at the University of Ottawa in Canada.[107]

The model approach also brings potential challenges and benefits to communicating the SDOH. They are particularly beneficial in that they depict the influence of social, economic, and political factors at multiple levels. Some models even identify areas where action on the SDOH can be taken.[15, 108] Others serve to illustrate pathways, which are helpful to individuals in understanding the ‘how’ behind the SDOH. One of the challenges is that they may oversimplify (and thus misrepresent) or overcomplicate (and thus overwhelm) the SDOH. To the extent that models and lists do not resonate with members of the public, for any reason, they may not instill a sense of need or urgency to act (e.g., contact their elected representative on SDOH-related matters), to the detriment of public engagement in public policy decision-making.

#### Communicating the SDOH through stories or narratives

The story, or narrative, approach to communicating the SDOH provides a way to convey feelings or experiences that simply is not possible in lists and models. A well-known example is the Public Health Agency of Canada’s narrative that asks the question, “Why is Jason in the hospital?”[109] The story goes on to answer increasingly structural questions about why Jason was in the hospital, had an infection, played in the junk yard, et cetera. Each answer reveals a wider set of social influences on Jason’s health. While the story approach did present itself in our review, it was not common. This is likely an artifact of our review methods, which did not include searching specifically for sources aimed at members of the public.

The story approach may fill the emotive void left by list and model approaches, to make the SDOH compelling to audiences. When reading an SDOH narrative, readers may experience feelings relating to luck, privilege, or fairness, and in some cases may even be compelled to act.[110] The story approach may lack the structure required to gain credibility in policy decisions when used on its own, though it may be more effective when used in combination with lists or models in its ability to convey complex information in understandable ways. A good illustration of this combined approach comes from the WHO CSDH final report, which includes a list (three overarching recommendations for action: [1] improve the conditions of daily life; [2] tackle the inequitable distribution of power, money and resources; and [3] measure and understand the problem and assess the impact of action [17]]; a conceptual framework, or model, of the SDOH, which allows readers to visualize how certain factors work together to influence health; and a story: the report itself is written in a way that crafts a compelling narrative, such as its use of case examples and personalization (e.g., “Social injustice is killing people on a grand scale.”[17]).

#### Epistemological differences in presenting the SDOH

The different ways of presenting the SDOH may align with the different epistemologies that underlie the literature. Ashcroft (2010) identifies objectivism, constructionism, and subjectivism as epistemologies present within the SDOH paradigm.[80] Objectivism was predominantly visible in articles that used statistical methods to explore and quantify the relationship between health and SDOH, where knowledge was produced in the context of epidemiology and population health.[80] Some examples include Regidor’s (2006) review of methods measuring socioeconomic position [24] and Gustafsson et al.’s (2014) quantitative analysis of the life-course accumulation of neighbourhood disadvantage and allostatic load.[79] There were also examples of a constructivist paradigm apparent in this review, where articles sought to present an understanding of the SDOH based on knowledge they had collected from the experiences of others. In their interviews with Medical Officers of Health and public health unit staff, Raphael, Brassolotto, and Baldeo (2014), for example, showed how public health professionals perceived the SDOH and how their role in acting on them differed, depending on whether or not their public health unit supported activities beyond traditional public health services or programs (e.g., vaccination, health education), such as advocacy on issues like hunger or poverty, or raising public awareness of the SDOH.[30] Finally, examples of subjectivism from this review are apparent in the articles that incorporated Aboriginal understandings and experiences of SDOH, through recognition of important influential factors such as the dispossession of land, cultural resilience, self-determination, and legacy of residential schools.[18, 66, 84, 108, 111–113]

We did not explore the literature with the explicit intent of discerning epistemologies, which limits our ability to comment on the extent to which different ones were used. However, in considering our sample at face value one thought is that academic authors may adopt more objectivist or constructivist epistemologies compared to non-academics, perhaps drawing on or developing their own conceptual models or frameworks to present their findings. This seems likely considering that non-academic authors may wish to convey the SDOH in ways more relatable and easily understood by a general audience or the public, and therefore may adopt more constructivist or subjectivist epistemologies through using lists and narratives.

### Health equity: A key theme of the SDOH

One theme emerged prominently during our analysis: health equity as an overarching theme and binding concept for the SDOH. We furthermore found that this binding concept of health equity was conceptualized in different ways, which align with more ‘upstream’ and ‘downstream’ orientations. We describe this observation in more detail below.

#### Health equity as a binding concept for the SDOH

The concept of health equity, which refers to a socially just state wherein all members of a population have access to the best available opportunities for health,[56] frequently appeared as an essential element in the SDOH. Health inequity, accordingly, refers to unfair and avoidable differences in health between population groups that reflect inequitable access to those opportunities.[17] Health equity and inequity are considered the socially produced results of systematic societal processes that contribute to distribution of resources.[114]

In the literature reviewed here, health equity was predominantly used when discussing the structural or societal-level changes needed to improve health. Studies also referred to health equity when making ethical claims (e.g., health equity as a normative concept, where a fair society is explicitly valued),[16] when discussing approaches to intervene on the SDOH (e.g., taking a targeted approach to intervention, that focuses on those living in disadvantaged circumstances),[115] and when discussing causes of ill health between social groups.[67]

Quantitative content analysis of all sources revealed the frequency of use of the above terms. Equity terms (equity, inequity, inequities) were used in 77.8% of articles (n = 84). These terms were employed most frequently in WHO-related documents; namely, the WHO CSDH final report and documents that referenced this report. Other papers that frequently used the term health equity included a sociological commentary on health equity,[69] an Alberta Health Services publication on social environments and health,[93] and a paper produced by a private organization regarding the SDOH agenda in Canada.[116] Terms related to equality (equality, inequality, inequalities) were used in 79.6% of articles (n = 86). Equality terms were used most frequently in two articles by Graham, in her writings on the SDOH in the context of UK government policy.[61, 103] Others included a research article comparing welfare state regimes,[33] and a commentary reviewing key tenets of the WHO CSDH.[117] The frequency of use for these terms is further broken down in Table 2.

**Table 2:** Counts of key terms used in the SDOH literature.

Key Term	No. (N = 108)	%
Equity, inequity, or inequities	84	77.8
Equity	76	70.4
Inequity or inequities	69	63.9
Equality, inequality, or inequalities	86	79.6
Equality	20	18.5
Inequality or inequalities	85	78.7
Social gradient	40	37.0
Social hierarchy	32	29.6
Social ladder	10	9.3
Social position	61	56.5

Related to health equity and inequity are the terms equality and inequality. Equality and inequality also refer to differences in health as present or absent, but do not carry the same moral undertones as equity and inequity.[114] They allow health differences to be acknowledged and discussed without necessarily adopting ethical or normative claims about what is fair and avoidable. As shown in Table 2, the terms (in)equity and (in)equality appeared with similar frequency. This may simply reflect that the terms are used interchangeably.[103] Alternatively, (in)equality may be intentionally employed to avoid the implications associated with the reasons for differences in health, from an SDOH point of view. Such strategic use of terms may be especially true for studies published by authors within organizations where there may be real or perceived consequences of associating SDOH-related differences as unfair (i.e., bearing political critique).[27] Finally, the use of (in)equity over (in)equality and vice versa may reflect differences in how understandings of the SDOH are conceptualized and how they operationalize different means of action (e.g., policy change, targeted health services). We expand on this third reason below.

#### The social gradient in health: A key concept of the SDOH

Another key concept we observed as prominent in the literature reviewed is the social gradient in health, which refers to the stepwise relationship between health and social position.[81, 118] According to the social gradient, which “runs right across society” health status is influenced by an individual’s position in the social hierarchy, which itself is influenced by social, political, and economic circumstances.[81, 118]

The social gradient in health appeared in various forms in the literature that we reviewed. For example, some articles attempted to quantify the social gradient (e.g., by measuring the relationship of occupational class and health).[81] Other articles included discussion on the chances individuals had to achieve good health.[111] Finally, some articles contained content that aligned strongly with the concept of the social gradient in health but using different language–a good example is the theory of fundamental causes (i.e., that the association between health and social status endures due to the access one has to fundamental, health protecting resources such as money, knowledge, and power).[95]

To further interrogate the use of the social gradient in health in the literature, we conducted a quantitative content analysis of the social gradient in health and related terms. As shown in Table 2, ‘social gradient’ was used in 37% of our articles (n = 40); ‘social hierarchy’ was used in 29.6% of articles (n = 32); ‘social position’ was used in 56.5% (n = 61); and, ‘social ladder’ was used in 9.3% (n = 10). These findings suggest the wide use of this concept.

### Conceptualizations of action on the SDOH towards health equity

While health equity was a common element of the literature we reviewed, (e.g., [17, 111, 119]), it was conceptualized in different ways. To describe the different conceptualizations, we draw on the concepts of upstream and downstream as well as the work of Graham (2004)[120] and Whitehead (2007).[121] Graham (2004), identifies the three policy approaches to tackling health inequalities: (1) remedy health disadvantage by improving the health of poor groups (e.g., policies that target poor groups); (2) narrow health gaps by improving the health of poor groups relative to other population groups (e.g., policies that improve the health of poor groups faster than other groups); and (3) reduce health gradients by tackling the root causes of illness (e.g., policies that redistribute wealth).[120] Whitehead (2007) provides an alternate but complementary typology for actions to reduce health inequalities, where she categorizes the aims of policies or interventions as: (1) strengthening individuals (e.g., health information campaigns or life skill groups); (2) strengthening communities (e.g., building neighbourhood meeting spaces to facilitate social inclusion); (3) improving living and working conditions (e.g., improving access to adequate housing); (4) promoting healthy macro-policies (e.g., ensuring healthy labour market policies).[121] Both typologies inform our discussion of ‘upstream’ and ‘downstream’ interventions on the SDOH.

Briefly, upstream interventions seek to diminish the ‘causes-of-the-causes’ of illness by acting on structural determinants of health that distribute wealth, power, and opportunities at the policy level.[23] According to the Nuffield Council on Bioethics’ ‘intervention ladder,’ which suggests that public health interventions affect people’s choices in more and less intrusive ways requiring more or less justification, policy interventions tend to occur higher up on the ladder (e.g., eliminate, restrict, or guide choice through incentives or disincentives).[122] Downstream interventions, on the other hand, act on the ‘effects of causes’ of illness by attempting to meet the immediate needs of individuals and families by improving their access to health and social services.[23] Often, downstream interventions focus on meeting the needs of certain population groups that face adverse health outcomes. On the ladder of intervention, downstream interventions tend to occupy lower rungs (e.g., enable choice, provide information, do nothing).[122]

Because the social gradient in health pertains to entire populations, it highlights the need for interventions that will tackle the distribution of health determinants.[120] As Graham (2004) explains, such interventions would involve structural-level policies (e.g., availability of housing for a range of incomes and life circumstances) that equalize life chances.[120] According to Whitehead’s (2007) typology for action on the SDOH, this is achieved through ‘upstream’ actions that aim to improve living and working conditions at a societal level, or by promoting macro-policies that address the SDOH at a structural level.[121] Graham’s reducing health gradients approach, and Whitehead’s category 4, align closely with the WHO CSDH’s recommendation to “tackle the inequitable distribution of power, money, and resources” through strengthening governance and the public sector, ensuring an accountable private sector, and leveraging health equity and collective action as issues of importance to the general public.[17]

#### Upstream action on the SDOH

Much of the literature that we reviewed supported an upstream approach to health equity by reducing gradients and promoting healthy macro-level policies. A major emphasis among articles supporting a gradient approach was the recognition that a collaborative and integrative approach would be necessary. Some articles called for greater collaboration between disciplines, departments, or sectors (e.g., academic disciplines, government departments, public/private).[15, 17, 22, 26, 64, 75, 89, 93, 116, 123–124] Others encouraged a ‘whole-of-society’ approach where citizens would mobilize themselves to bring change to societal conditions in ways that facilitate health equity, at times transcending the health sector to areas such as education, social welfare, food and drug administration, et cetera.[17, 20, 26, 71, 78, 87, 96, 116]

A good illustration of the gradient and a healthy macro-policy approach to acting on the SDOH is Brennenstuhl et al.’s (2012) systematic review of welfare regimes and population health inequalities.[125] The authors found that a social democratic approach to governance, whereby social and economic interventions that support equality are implemented within a capitalist framework, with policies such as generous pensions, fostered population health and health equity (e.g., lower infant mortality rate).[125] It is important to note that empirical evaluative research on this social democratic approach to governance is limited.

While upstream, gradient, and healthy macro-social policy approaches seem desirable for their ability to address the root causes of health inequities and act on multiple SDOH across sectors,[17] they have also been subject to criticism. Popay et al. (1998), for instance, reviews three critiques of quantitative research on inequalities in health: (1) they may lack explanatory power in linking agency and structure relating to health inequalities, in the context of social structures, (2) macrosocial frameworks may fail to address complexities in explaining the causes of health inequalities, and, (3) the notion of place, as defined in macrosocial explanatory models, has not been conceptualized in its social and historical contexts (e.g., class, living place, gender, age, et cetera).[126] The critical population health research perspective, which “requires asking more critical questions about the social and economic causes and consequences of health inequalities…”[127] appears to have been one response these critiques that has gained momentum in recent research on SDOH (e.g., Richmond and Ross 2009; Labonté, Polanyi, Muhajarine, et al. 2005).[2, 18]

#### Action on the SDOH further downstream

In other articles, action on the SDOH was understood with reference to specific subgroups facing social disadvantage. This approach to ‘tackling health inequalities’) can be considered more ‘downstream.’[103] That is, rather than addressing the wider structural (i.e., social, political, economic) influences that shape the distribution of health determinants, this approach focused more on meeting the immediate needs of individuals adversely affected by health inequality, such as by increasing access to services.[103] This conceptualization aligns with the ‘remedying health disadvantages’ approach presented in Graham’s (2004) typology of tackling health inequalities.[120] and to approaches that, according to Whitehead (2007), involve attempts to strengthen individuals (category 1) or communities (category 2) characterized by socio-economic disadvantage, for example by developing the life skills of or building social cohesion in these communities.[121] As Graham (2004) notes, one drawback of a remedying health disadvantages approach is that it may narrow the scope of potential policy solutions to those that focus on individuals experiencing social disadvantage (i.e., a targeted approach), which may be less effective if action on broader determinants that create social disadvantage is not considered.[120] Furthermore, intervention approaches that are confined to sub-groups (i.e., ‘those at the bottom’), may bring negative effects to other groups, such as the potential stigmatization that may occur when publicly identifying a vulnerable group for the purpose of intervention.[120]

In some cases, as noted by Frohlich et al. (2006), an approach focusing on disadvantaged sub-populations is necessary where certain groups may require special attention at the community level so not to be adversely affected by certain policies.[112, 128] As some authors, including Graham, have acknowledged, there is a strong moral basis for tackling health inequalities at this level (especially in wealthy nations) so that those at the bottom are not denied of their basic needs or the average standard of living enjoyed by the majority of the population.[120] In certain circumstances (e.g., where people experience ill health or disability as the result of social, political, and economic inequalities), it may be necessary to focus on improving the health of those most disadvantaged so they do not fall further behind the rest of the population. The notion of *proportionate universalism* has been put forth to recognize the challenges posed by population-level interventions for certain groups.[129–131] The idea behind proportionate universalism, as stated in *Fair Society*, *Healthy Lives*: *The Marmot Review*, is, “To reduce the steepness of the social gradient in health, actions must be universal, but with a scale and intensity that is proportionate to the level of disadvantage.”[129]

While proportionate universalism was not prominent in our target literature (which makes sense considering its recency), we found that targeted and downstream approaches to health equity and the SDOH were prevalent in the literature. In many cases this reflected the professional scope of the authors. The Canadian Nurses Association, for example, put forth an idea for how individual nursing practice could act on the SDOH, by asking patients certain questions (e.g., their circumstances relating to income or housing), considering whether patients have access to health resources or recommended medical treatments, or knowing available community resources that could assist their clients.[91] Other articles spoke of physicians’ “ethical duty to their patients” to act on the SDOH,[92] or the need for physicians to “be the natural attorneys of the disadvantaged.”[75]

A similar trend towards targeted and downstream approaches to action on the SDOH was found in literature reporting on the public health workforce McIntyre et al.’s (2013) focus group with individuals who were affiliated with Canadian public health, for example, found that many participants were too preoccupied with behavioural and lifestyle approaches (e.g., diet, exercise), to attempt action at broader levels that may tackle wider spheres of influence on the determinants of health (e.g., neighbourhood, environment).[132] This may in part reflect challenges identified by Raphael et al. (2014) in their work with public health units, where participants identified that they “act where [they] think that there’s some prospect of actually changing something”; namely, through the provision of downstream services and programs.[29] In a related paper by Raphael’s group, Brassolotto et al. (2014) found that public health practitioners predominantly considered the SDOH as things to be mindful of in practice but that occurred outside the scope of their work.[27, 30] One participant stated that, “It may be emotionally satisfying to think that we can go out and restructure Canadian society. It’s self-indulgent, in my opinion, and it’s not the business we’re in.”[27] Finally, in a paper contemplating how social theory could be integrated into public health practice Potvin et al. (2005) found that public health practitioners in Canada faced difficulties acting on the SDOH because of the bureaucratic nature of their practice and its lack of instruments to implement innovative practices.[133]

An emphasis on downstream approaches to remedying health inequities may also apparent in academic scholarship. Raphael and Bryant (2015) have asserted that, aside from a handful of critical scholars, academics predominantly do not write about action on the SDOH in ways that focus on upstream, macrosocial factors in their research.[134] Unlike the public health practitioners who face potential consequences in their place of employment, however, these authors state that “the academics don’t have that excuse […] especially the tenured ones.”[134]

In summary, in the literature reviewed we identified that though health equity is a common, binding concept in the SDOH, there are different ways in its conceptualization. These differences have implications for approaches to action, which range from a more upstream, structural approach that considers the social gradient as well as the determinants and processes that distribute resources across the population, to a more downstream, community or individual-level approach that focuses on social or behavioural factors operating within specific groups (sometimes, but importantly not always, these social and behavioral factors are considered discretely or in isolation). The different approaches do not appear to have been strongly reconciled.

## Limitations

The main limitations of this scoping review are threefold. First, our internet (Google) search for SDOH literature was conducted from a Canadian Internet Protocol address, which may have returned results specific to our geographic setting (Canada) and thus inflated the estimates of Canadian content. This may represent Canada’s ‘policy strong’ reputation on the SDOH that exists in writing, but not in its government’s actions, as noted earlier. Future work may consider comparing how grey literature on the SDOH in other countries differs from that produced in Canada. Another facet of this limitation is that our search for grey literature utilized Canadian databases (i.e., Canadian Research Index, Canadian Health Research Collection), which potentially over-represented literature from this setting. However, the academic literature revealed similar proportions as found for grey literature (i.e., Canada, followed by United States and United Kingdom), which suggests this may not be the case. As well, despite this limitation, our inclusion of grey literature still adds novelty and merit to existing literature reviews on the SDOH.

Second, our search included “social determinants of health” as a search term. To the extent that other countries or disciplines discuss the concept of SDOH using different language (e.g., ‘social factors’ instead of social determinants), we may have missed important content. Our use of “public health,” “population health,” and “health promotion” and searching different disciplinary databases should have offset this limitation to some extent. Furthermore, we attempted to address this limitation through our iterative and nuanced approach to the search (i.e., not simply relying on the presence/absence of terms, see Fig 1. Visual representation of approach to inclusion criteria), which we argued was essential for this literature because of its somewhat diffuse and jargonized nature.

## Conclusion

In this scoping review, we set out to take stock of and synthesize SDOH literature in the contemporary context of population and public health. Our main conclusions are threefold. First, most of the literature has been published in the last decade (2005–2009), in academic settings, with the intent of reaching policy makers. This likely reflects the impact of the WHO CSDH on the population and public health community. Just over half of the literature came from Canadian sources. Second, the SDOH were communicated in three ways as a list, conceptual model or framework, or narrative or story. Each form of communication appears to have met the needs of different authors and audiences. To some extent, these forms of communicating the SDOH may have aligned with the epistemologies of objectivism, constructivism, and subjectivism. Third, we identified health equity as a binding concept and overarching theme of the SDOH. In part, this was observed in the literature through the frequent use of key terms related to health equity, such as the social gradient in health. We also found that different ways of achieving health equity, through action on the SDOH, were conceptualized as more upstream or downstream in nature. Overall, we found that the current literature did not unanimously adopt the language of health equity when presenting and discussing the SDOH. We suggest that intentional articulation of the SDOH in this way by authors may help unify the message that the SDOH are fundamentally about health equity.

This review has identified a literature gap for articles published from countries outside the global north. Very few authors were situated in developing or poor countries, which limits our understanding of the SDOH at a global level and the transferability of our findings. This is especially important considering that recent work by the WHO has called for the global redistribution of resources to achieve health equity worldwide.[17] It is imperative that countries adversely affected by international decisions regarding the distribution of economic resources have a seat at the table with the countries holding social, economic, and political power; this is important in recognition of global justice and fairness in relations between wealthy and poor nations. We encourage future contributions to the SDOH literature from those working in PPH in developing nations.
